# Acute Microbial Protease Supplementation Increases Net Postprandial Plasma Amino Acid Concentrations After Pea Protein Ingestion in Healthy Adults: A Randomized, Double-Blind, Placebo-Controlled Trial

**DOI:** 10.1016/j.tjnut.2024.03.009

**Published:** 2024-03-11

**Authors:** Kevin J.M. Paulussen, Andrew T. Askow, Max T. Deutz, Colleen F. McKenna, Sean M. Garvey, Justin L. Guice, Richard M. Kesler, Takeshi M. Barnes, Kelly M. Tinker, Scott A. Paluska, Alexander V. Ulanov, Laura L. Bauer, Ryan N. Dilger, Nicholas A. Burd

**Affiliations:** 1 Department of Health and Kinesiology, University of Illinois Urbana-Champaign, Urbana, IL, United States; 2 Division of Nutritional Sciences, University of Illinois Urbana-Champaign, Urbana, IL, United States; 3 Department of Research and Development, BIO-CAT, Inc., Troy, VA, United States; 4 Illinois Fire Service Institute, University of Illinois Urbana-Champaign, Urbana, IL, United States; 5 Roy J. Carver Biotechnology Center, University of Illinois Urbana-Champaign, Urbana, IL, States; 6 Department of Animal Sciences, University of Illinois Urbana-Champaign, Urbana, IL, United States; 7 Beckman Institute for Advanced Science and Technology, University of Illinois Urbana-Champaign, Urbana, IL, United States

**Keywords:** plant-based protein, anabolism, leucine, protein foods

## Abstract

**Background:**

Digestibility is a primary factor in determining the quality of dietary protein. Microbial protease supplementation may be a strategy for improving protein digestion and subsequent postprandial plasma amino acid availability.

**Objectives:**

To assess the effect of co-ingesting a microbial protease mixture with pea protein on postprandial plasma amino acid concentrations.

**Design:**

A mixture of 3 microbial protease preparations (P3) was tested for proteolytic efficacy in an in vitro static simulation of gastrointestinal digestion. Subsequently, in a randomized, double-blind, placebo-controlled crossover trial, 24 healthy adults (27 ± 4 y; 12 females, 12 males) ingested 25 g pea protein isolate (20 g protein, 2.2 g fat) with either P3 or maltodextrin placebo (PLA). Blood samples were collected at baseline and throughout a 0‒5 h postprandial period and both the early (0–2 h) iAUC and total (0–5 h) iAUC were examined.

**Results:**

Plasma glucose concentrations decreased in both conditions (*P* < 0.001), with higher concentrations after P3 ingestion compared with PLA (*P* < 0.001). Plasma insulin concentrations increased for both conditions (*P* < 0.001) with no difference between conditions (*P* = 0.331). Plasma total amino acid (TAA) concentrations increased over time (*P* < 0.001) with higher concentrations observed for P3 compared with PLA (*P* = 0.010) during the 0‒5 h period. There was a trend for elevated essential amino acid (EAA) concentrations for P3 compared with PLA (*P* = 0.099) during the 0‒5 h postprandial period but not for leucine (*P* = 0.282) or branched-chain amino acids (BCAA, *P* = 0.410). The early net exposure (0‒2 h iAUC) to amino acids (leucine, BCAA, EAA, and TAA) was higher for P3 compared with PLA (all, *P* < 0.05).

**Conclusions:**

Microbial protease co-ingestion increases plasma TAA concentrations (0–5 h) and leucine, BCAA, EAA, and TAA availability in the early postprandial period (0‒2 h) compared with ingesting pea protein with placebo in healthy adults.

## Introduction

Dietary protein ingestion leads to an increase in postprandial amino acids in circulation and the stimulation of whole body and muscle protein synthesis [[1], [2], [3]]. Diet-, age-, and exercise-associated changes in postprandial aminoacidemia have been shown to modulate the anabolic response to protein consumption [[4], [5], [6]]. The digestibility of a given protein source is a key factor in determining kinetics for postprandial release of protein-derived amino acids into circulation. For example, previous studies have shown decreased digestion and absorption rates from plant-derived dietary protein sources such as soy, pea, and wheat [[7], [8], [9]] compared with higher quality animal-based protein sources such as whey, beef, and milk [5,10,11]. A commonly suggested strategy to overcome the limited digestibility of a protein source is to simply increase the amount of protein ingested to increase plasma amino acid availability [12]; albeit this strategy of eating excessive amounts of plant-protein whole foods to augment anabolic potential appears to be ineffective [11]. Alternatively, the postprandial rise in plasma amino acid concentrations could potentially be improved through co-ingestion with supplemental digestive enzymes, specifically proteases.

Microbial proteases have been investigated as a nutritional strategy to enhance the postprandial rise of amino acid concentrations in the circulation after protein ingestion [13,14]. Importantly, 3 in vitro studies using the INFOGEST static or semidynamic simulation of gastrointestinal (GI) digestion have demonstrated that microbial proteases obtained from *Aspergillus* spp. enhance the digestion of protein from a variety of sources including pea, dairy, collagen, soy, wheat, and chicken meat [[15], [16], [17]]. However, in vivo human studies have demonstrated mixed results when attempting to translate the positive outcomes of in vitro studies [13,14]. For example, Oben et al. [13] demonstrated increased postprandial aminoacidemia when a fungal protease blend was co-ingested with 50 g whey protein concentrate. Conversely, Townsend et al. [14] reported no postexercise effect on plasma amino acid concentrations following co-ingestion of a mixture of a bacterial protease and the plant protease bromelain with 26 g whey protein. Considering these differential outcomes, additional human studies are needed to help clarify the effects of acute protease co-ingestion on dietary protein digestibility and postprandial aminoacidemia. Such information will help define more effective performance and clinical nutrition feeding strategies to augment the anabolic potential of dietary protein, especially less digestible proteins such as those from plant-derived sources.

Our first aim was to identify an optimal mixture of microbial proteases for dietary protein digestion in the INFOGEST in vitro static simulation of GI digestion. Next, we aimed to translate these in vitro findings into a human clinical intervention trial to investigate the effects of protease and pea protein co-ingestion on postprandial rise in plasma amino acid concentrations in healthy adults. Pea protein isolate was selected due to its commercial relevance as a plant-based protein source (i.e., sufficient availability and cost-effectiveness). In addition, through in vitro testing, we demonstrated that microbial proteases more effectively increased amino acid release from pea protein compared with whey protein (described herein). We hypothesized that co-ingesting pea protein isolate with microbial proteases would result in augmented postprandial plasma amino acid concentrations compared with pea protein isolate with placebo (PLA). We also examined subjective appetite and GI measurements for additional insights into the impact of protease supplementation on GI symptoms, satiety, and appetite.

## Methods

### Investigational products

The following 3 nongenetically engineered, wild-type microbial enzyme preparations are distributed by BIO-CAT, Inc.: Fungal Protease A2 obtained from *Aspergillus oryzae* [Chemical Abstracts Service (CAS) No. 9074-07-1], Fungal Protease A obtained from *A. oryzae* (CAS No. 9025-49-4), and Protease AM obtained from *A. melleus* (CAS No. 9074-07-1). These individual enzyme preparations contain diluents (i.e., potato dextrin or tapioca maltodextrin) and are standardized on proteolytic activity, expressed as hemoglobin units on the tyrosine basis (HUT). One HUT unit of proteolytic (protease) activity is defined as the amount of enzyme that, in 1 min under the specified conditions, produces a hydrolysate from bovine hemoglobin at pH 4.7 whose absorbance at 275 nm is the same as that of a solution containing 1.10 μg·mL^-1^ tyrosine in 0.006 N hydrochloric acid [18]. OPTIZIOME® P³ HYDROLYZER® (hereafter referred to as “P3”; BIO-CAT, Inc.) is a powder mixture of the 3 abovementioned microbial protease preparations formulated to contain 31,875 HUT units per 250 mg dose. Maltodextrin from waxy maize (Cargill Inc.) was included in P3 as a diluent. For the in vitro simulations of GI digestion, P3 powder (Lot No. OZP#H-LE19-B) was tested. For the clinical trial, P3 (Lot No. OPTIP3H-LE08) was manufactured within size 1 cellulose capsules (Summit Lake Labs, LLC). Each P3 capsule was formulated to contain 31,875 HUT (without overage) and 20 mg microcrystalline cellulose (MCC, flow agent) for a total weight of 270 mg per capsule. PLA capsules were manufactured with 250 mg maltodextrin and 20 mg MCC. Pea protein isolate (Lot No. W486N, NUTRALYS® S85F pea protein isolate, Roquette Frères) was used as the substrate for both the in vitro GI digestion simulations and the clinical trial. The manufacturer’s report of the nutrient content of the pea protein was confirmed by proximate and amino acid analysis according to methods set forth by the Association of Official Analytical Collaboration [19] through an independent laboratory (Table 1). Whey protein concentrate (Lot No. 3190436, Avonlac® 282GLC, Glanbia) was included as an additional substrate for the in vitro digestion simulations.

**TABLE 1:** Nutrient content of the pea protein isolate

Nutrient	100 g	25 g
Energy (kcal)^1^	405	101
Moisture (g)^2^	6.33	1.58
Crude fat (g)^3^	8.98	2.25
Carbohydrates (g)^1^	0.84	0.21
Crude protein (g)^4^	79.96	20.00
Total amino acids (g)^5^	79.40	19.85
Amino acids (g)^6^		
Alanine	3.33	0.83
Arginine	6.89	1.72
Aspartic acid/asparagine	9.10	2.28
Cyst(e)ine	0.80	0.20
Glutamine/glutamic acid	14.31	3.58
Glycine	3.18	0.80
Histidine	1.98	0.50
Isoleucine	4.07	1.02
Leucine	6.63	1.66
Lysine	6.07	1.52
Methionine	0.73	0.18
Phenylalanine	4.41	1.10
Proline	3.39	0.85
Serine	3.80	0.95
Threonine	2.79	0.70
Tryptophan^7^	0.75	0.19
Tyrosine	2.89	0.72
Valine	4.28	1.07

1 Calculated.

2 Determined via AOAC 925.10.

3 Determined via AOAC 922.06.

4 Determined via AOAC 990.03.

5 Calculated as the sum of determined amino acid concentrations.

6 Determined via AOAC 982.30 E(a,b,c) unless otherwise noted.

7 Determined via AOAC 988.15.

### In vitro digestion simulations

The INFOGEST in vitro static simulation of GI digestion was used to test the efficacy of the 3 microbial protease preparations individually, and in combination (P3), on pea and whey protein digestion. The international consensus INFOGEST protocol has been described elsewhere [20], including an adaptation of the protocol for the study of supplemental enzymes [16]. Briefly, the INFOGEST protocol was used to simulate 3 phases of digestion—oral, gastric, and small intestinal—in 250 mL beakers. The oral phase proceeded for 2 min in a simulated salivary fluid with agitation by magnetic stirring at 37°C at a neutral pH in the presence of porcine salivary amylase. The gastric phase was initiated by the addition of simulated gastric fluid containing porcine pepsin and adjustment of the pH to 3, followed by 2 h of stirring at 37°C. The intestinal phase was initiated by the addition of a simulated intestinal fluid containing porcine pancreatin and bile extract and adjustment of the pH to 7, followed by 2 h of stirring at 37°C. In the protease treatment conditions, a partial 1/30th dose of individual microbial proteases or P3 was prepared in 1 mL deionized water and combined with a 9 mL solution containing 1 g pea or whey protein (equivalent to ∼1/30th a serving size). Control conditions included protein substrate without supplemental microbial enzymes. Following the 2 h gastric phase, 10 mL samples of digesta were collected to examine proteolytic activity under oro-gastric conditions. Digesta samples were also collected after the 2 h intestinal phase to test proteolytic activity across the full oro-gastrointestinal simulation. All withdrawn samples were immediately heat-inactivated at 90°C for 10 min and frozen at –20°C. Experiments were performed in triplicate on separate days. For amino acid analysis, digesta samples were thawed, mixed well, and filtered through a 0.2-μm polyvinylidene fluoride syringe filter prior to dilution in 0.1 N HCl. Gastric digesta samples were analyzed as is, and intestinal digesta samples were analyzed at a 1:10 dilution. Digesta amino acid concentration was measured by high-performance liquid chromatography (Agilent 1100 Series HPLC, Agilent Technologies, Inc.) with fluorescence detection using an established method [16]. Results are reported in milligrams amino acid per gram starting protein substrate powder.

### Clinical trial

#### Study design and ethical approval

A randomized, double-blind, placebo-controlled, crossover study of healthy, recreationally active, young adults was conducted from April 2021 to August 2021 at a single research site (University of Illinois Urbana-Champaign) to test the acute effects of P3 on postprandial plasma amino acid concentrations after consuming pea protein. The clinical trial consisted of an online screening visit, an in-clinic secondary screening visit, and 2 in-clinic treatment visits. All visits conformed to standards for the use of human participants in research as outlined in the Helsinki Declaration, and all study procedures were reviewed and approved by the Institutional Review Board at the University of Illinois Urbana-Champaign (IRB No. 21545). The study was prospectively registered at ClinicalTrials.gov (NCT04821557).

#### Participants

Twenty-four recreationally active, healthy adults (12 female, 12 male; 24.8 ± 1.9 kg·m^-2^; 27 ± 4 y) volunteered to participate in this study. The flow of participants through the intervention is depicted in Supplemental Figure 1. All participants were deemed healthy and physically active based on their responses to both a routine medical screening questionnaire and the International Physical Activity Questionnaire (IPAQ) completed during an online screening. All participants were informed about the experimental procedures, the purpose of the study, and potential risks before providing informed consent. Following the completion of preliminary online screening, participants underwent an in-clinic secondary screening visit 2 wk before the first in-clinic treatment visit. During this screening, participants reported to the laboratory in the morning following an overnight fast for measurement of body mass, height, and body composition via dual-energy X-ray absorptiometry (DEXA; Horizon W, Hologic Inc.,). Upon confirmation of eligibility, participants were randomly assigned and counterbalanced for enrollment. Female participants completed their visits during the follicular phase of the menstrual cycle to lessen any impacts on baseline plasma amino acid concentrations [21]. A computer-generated list of random numbers was used for the allocation of participants. The study product was coded with a random computer-generated number and kept blinded until data collection and analysis were completed.

#### Study procedures

**FIGURE 1:**
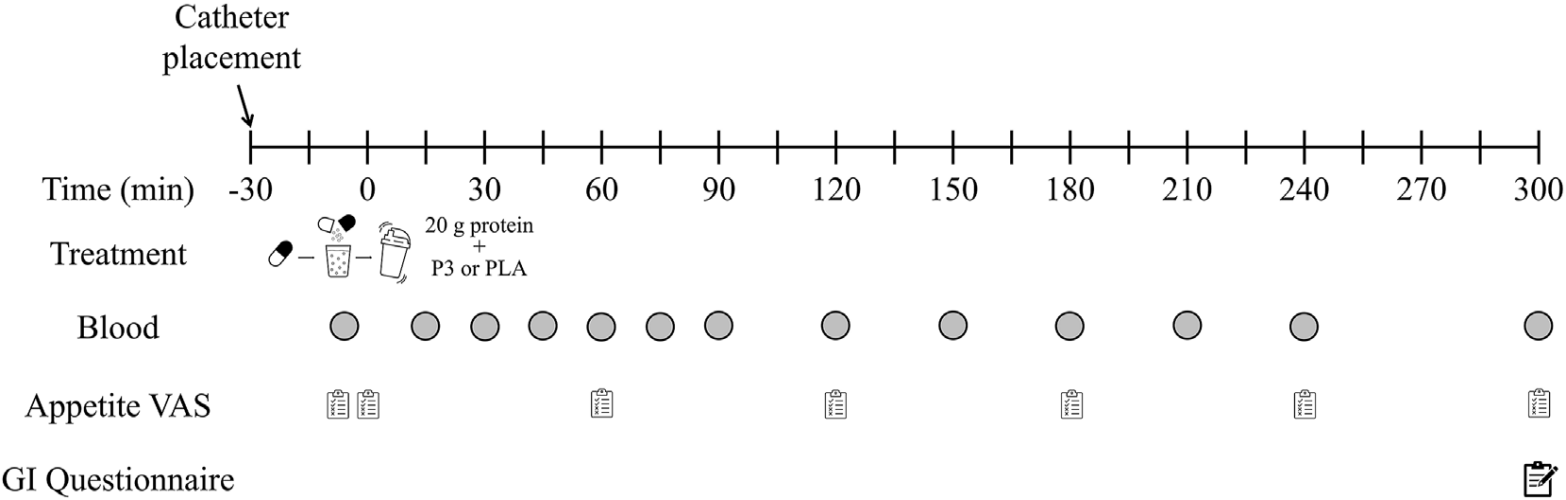
Schematic of the experimental design for the clinical trial in this randomized, double-blind, placebo-controlled crossover study. GI, gastrointestinal; P3, mixture of 3 microbial protease preparations; PLA, placebo; VAS, visual analog scale.

An overview of the experimental protocol during each treatment visit is shown in Figure 1. Participants were instructed to refrain from any strenuous physical exercise or alcohol consumption for 72 and 48 h, respectively, prior to each treatment visit. A nutrient-density matched standardized meal was provided to participants for consumption the evening before each treatment visit (25%‒30% of energy requirement; 50% of energy from carbohydrates, 25% energy from protein, and 25% energy from fat). In addition, participants were instructed to maintain the same dietary intake for 3 d prior to each treatment visit. Dietary intake was tracked and confirmed using the Automated Self-Administered 24-hour (ASA24®) Dietary Assessment Tool (version 2020; National Cancer Institute).

On the morning of each of the 2 treatment visits, participants reported to the laboratory between 06:00 and 08:00 after an overnight fast. Following the assessment of blood pressure, an intravenous catheter was inserted into an antecubital vein and an arterialized baseline blood sample (*t* = –5 min) was collected. Immediately after the catheter placement and blood sample collection, participants ingested 25 g pea protein isolate (101 kcal, 20 g protein, 2.2 g fat) with either P3 or PLA. The contents of either study product capsule were emptied, dissolved, and thoroughly mixed with 300 mL water and the pea protein isolate within 5 min of being given to participants. Participants were allowed to drink water ad libitum throughout the first treatment visit, with equal volumes being provided during the second treatment visit. Additional arterialized blood samples were collected in EDTA-containing tubes after study product ingestion (*t* = 15, 30, 45, 60, 75, 90, 120, 150, 180, 210, 240, and 300 min). Blood samples were centrifuged at 3000 × *g* for 10 min at 4°C and the plasma was subsequently aliquoted and stored at –80°C. Three visual analog scales (VAS; [22]) for measurement of appetite sensations were administered at baseline, after protein ingestion, and subsequently every hour (*t* = –5, 0, 60, 120, 180, 240, 300 min). VAS were completed via digital sliding scales whereby the slider started halfway between the minimum score [i.e., farthest left; 0 AU (arbitrary units)] and maximum score (i.e., farthest right; 100 AU), and participants moved the slider to the position, which most reflected their subjective feelings of each appetite sensation. A GI symptom questionnaire was collected at the end of each trial day [23]. The washout period between treatment visits was ≥1 wk in duration. Each treatment visit of this crossover study was conducted identically except for utilizing the alternative study product during the treatment visit.

#### Biochemical assessments

##### Plasma glucose and insulin

Plasma glucose concentrations were analyzed by an automated biochemistry analyzer (2900 Stat Plus, YSI Life Sciences). Plasma insulin concentrations were determined using a commercially available ELISA (ALPCO).

##### Plasma amino acids

The amino acid standard solution (Product No. AAS18, MilliporeSigma), containing a combination of 2.5 μmol·mL^-1^ of L-alanine, L-arginine, L-aspartic acid, L-glutamic acid, glycine, L-histidine, L-isoleucine, L-leucine, L-lysine·HCl, L-methionine, L-phenylalanine, L-proline, L-serine, L- threonine, L-tyrosine, and L-valine; a concentration of 1.25 μmoL·mL^-1^ L-cystine; and a custom mixture containing 2.5 μmol·mL^-1^ of L-tryptophan, L-glutamine, L-asparagine, and L-cysteine, respectively, were utilized for the calibration curve in the quantification of plasma amino acid concentrations. Plasma samples (50 μL) were deproteinized with methanol (940 μL), centrifuged with the following supernatant evaporation in vacuum, and resuspended in 1 mL of 0.1% formic acid in water before instrument injection. Ten microliters of internal standard (DL-p-chlorophenylalanine, 1 mg·mL^-1^ 0.1 M HCL) was added to each sample and standard solution. Samples were then centrifuged, and the resulting supernatant was dried under vacuum before resuspension in 1 mL 0.1% formic acid in water prior to injection. Samples were analyzed by LC–MS–MS (Altis Triple Quadrupole, Thermo Fisher Scientific Inc.) with TraceFinder 4.1 data acquisition software. The LC separation was performed on a Thermo Accucore Vanquish C18+ column (2.1 × 100 mm, 1.5 μm) with mobile phase A (0.1% formic acid in water) and mobile phase B (0.1% formic acid in acetonitrile) at a flow rate of 0.2 mL·min^−1^. The linear gradient was as follows: 0‒0.5 min, 0% B; 0.5‒3.5 min, 60% B; 3.5‒5.5 min, 100% B; 5.5‒7.5 min, 0% B. The autosampler and HPLC column chamber were set at 10°C and 50°C, respectively. The injection volume was 1 μL. Mass spectra were acquired under positive electrospray ionization with the ion spray voltage of 3500 V. Selected reaction monitoring was used for the amino acid quantitation.

### Statistical analyses

#### In vitro digestion simulations

All in vitro data are presented as mean ± SD. A 1-way ANOVA was performed for all dependent variables. Model assumptions were tested by the Shapiro–Wilk test on residuals, Levene’s test of equality of variances, and visual inspection of residual and Q–Q plots. Violations of the model of assumptions were assessed for outliers with influence diagnostics. Comparisons were considered statistically significant if the ANOVA *F*-test *P* value was ≤ 0.05. Multiple pairwise comparisons were assessed with a post hoc Tukey's test (family-wise error rate = 0.05).

#### Clinical trial

##### Sample size calculation

An a priori power analysis for the human clinical trial was conducted using GPOWER version 3.1.9.2 [24]. Based on previous research [25], a power analysis showed that a sample size of 20 participants was sufficient to detect differences in postprandial iAUC BCAA concentrations between conditions when using a 1-tailed *t*-test (*P* < 0.05, 85% power, *d* = 0.62). Accounting for a potential dropout rate of 15%, we recruited a total of 24 participants.

##### Efficacy and ancillary assessments

Clinical trial data were assessed for normality via visual inspection of normal Q–Q plots and skewness/kurtosis values. Differences in blood glucose, insulin, and amino acid concentrations and appetite VAS results were analyzed using linear mixed-effects models (2-tailed) with time and condition as fixed factors and participant intercept as a random effect. Bonferroni’s post hoc test was used when significant main effects were identified. Differences in baseline dietary intake in addition to plasma glucose, insulin, amino acid iAUC, and amino acid *C*_max_/*T*_max_ values were analyzed using a 2-tailed paired samples *t*-test. Postprandial incremental area under the curve (iAUC) was calculated as described and adapted from Brouns et al. ([26], see Supplemental Methods). We calculated iAUC for both the early (0‒2 h) and total (0‒5 h) postprandial periods. We chose the 0‒2 h period to designate the early timeframe as this represents the typical time frame of peak amino acid availability [27,28]. Moreover, this period is a physiologically relevant postexercise window from a performance nutrition perspective to support the skeletal muscle adaptive response [29]. The McNemar test was used to examine differences in GI symptoms questionnaire responses between treatments. The level of statistical significance was set at *P* < 0.05 for all analyses. All analyses for human clinical trial data were performed using IBM SPSS Statistics 28.0 (IBM Corporation). All in vivo data are presented as mean difference (95% CI).

##### Imputation

Any missing amino acid concentration values were imputed using multiple imputations with the R Amelia (1.8.0) package [30]. The imputation model was fit using a cubic polynomial effect of time, including lag and lead times. Ten imputation iterations were performed for each amino acid. Imputations were visually inspected using a time-series cross-section plot for no effect, linear, squared, and cubic effects of time. Imputations were then assessed for plausibility using imputation diagnostics. Density comparisons were performed to compare imputation values to observed values. Model fitness was assessed using an overimputation diagnostic plot. Missing values were considered missing completely at random and accounted for <2% of a complete data set for each amino acid analyzed.

## Results

### In vitro digestion simulations

The efficacies of the P3 mixture of microbial proteases and its individual component proteases were confirmed via static in vitro simulations of pea and whey protein digestion. Following oro-gastric simulation of pea protein digestion, on average across all protease treatments, gastric digesta leucine, BCAA, EAA, and TAA concentrations were increased by 275%, 193%, 65%, and 78%, respectively, compared with the control group (all *P* < 0.0001, Table 2). Fungal Protease A treatment predictably resulted in less amino acid release than Fungal Protease A2 and Protease AM, which historically exhibit greater exopeptidase activity, including leucine aminopeptidase activity [16]. Following oro-gastric simulation of whey protein digestion, on average across all protease treatments, leucine, BCAA, EAA, and TAA were increased by 77% (*P* = 0.0003), 86% (*P* = 0.0003), 63% (*P* = 0.0007), and 56% (*P =* 0.0019), respectively, compared with the control group (Table 2). Fungal Protease A2 and P3 consistently showed greater proteolytic activity than Protease AM and Fungal Protease A. These data are consistent with the composition of P3, which contains Fungal Protease A2, Fungal Protease A, and Protease AM in order of descending inclusion rate. Following the oro-gastrointestinal simulations, amino acid concentrations of intestinal digestas showed no significant differences between groups (all *P* > 0.5302) (Table 2).

**TABLE 2:** Amino acid concentrations of gastric and intestinal digestas following simulated digestion of pea and whey proteins^1^

Substrate type	Control	Fungal Protease A2	Fungal Protease A	Protease AM	P3	ANOVA (F)
Digesta type						*P* value
Amino acid, mg·g^-1^						
Pea protein isolate						
Gastric						
Leucine^2^	0.50 ± 0.04^a^	2.09 ± 0.26^bc^	1.53 ± 0.24^b^	1.92 ± 0.24^b^	1.96 ± 0.03^bc^	<0.0001
Total BCAA^2^	0.77 ± 0.04^a^	2.51 ± 0.27^bc^	1.87 ± 0.30^b^	2.32 ± 0.27^bc^	2.32 ± 0.03^bc^	<0.0001
Total EAA^2^	3.70 ± 0.31^a^	6.80 ± 0.75^b^	5.32 ± 1.02^ab^	6.00 ± 0.71^b^	6.24 ± 0.25^b^	<0.0001
Total amino acids^2^	6.93 ± 0.47^a^	13.55 ± 1.27^c^	10.63 ± 1.48^b^	12.23 ± 1.11^bc^	12.91 ± 0.28^bc^	<0.0001
Intestinal						
Leucine	62.0 ± 9.2	64.3 ± 12.5	60.0 ± 4.7	59.1 ± 6.2	62.1 ± 3.9	0.9360
Total BCAA	110.4 ± 16.1	114.6 ± 22.2	107.0 ± 8.1	105.1 ± 11.1	110.4 ± 7.5	0.9340
Total EAA	263.4 ± 32.0	273.2 ± 50.9	253.8 ± 17.7	250.2 ± 25.3	264.8 ± 14.8	0.8942
Total amino acids	574.7 ± 50.3	585.3 ± 99.4	546.0 ± 35.0	541.0 ± 46.9	565.3 ± 27.9	0.8594
Whey protein concentrate						
Gastric						
Leucine^2^	1.35 ± 0.18^a^	2.68 ± 0.35^c^	2.21 ± 0.18^bc^	1.95 ± 0.09^ab^	2.71 ± 0.33^c^	0.0003
Total BCAA^2^	1.66 ± 0.21^a^	3.43 ± 0.47^bc^	2.88 ± 0.24^bc^	2.52 ± 0.10^ab^	3.54 ± 0.49^c^	0.0003
Total EAA^2^	5.00 ± 0.44^a^	8.72 ± 0.69^b^	7.43 ± 0.74^b^	7.18 ± 0.75^ab^	9.31 ± 1.28^b^	0.0007
Total AA^2^	8.03 ± 0.75^a^	13.31 ± 1.13^b^	11.56 ± 1.11^b^	11.28 ± 1.35^ab^	14.06 ± 1.90^b^	0.0019
Intestinal						
Leucine	78.0 ± 5.3	75.6 ± 6.1	79.4 ± 9.9	73.5 ± 4.9	72.2 ± 3.5	0.6115
Total BCAA	137 ± 9.9	133 ± 10.9	142 ± 17.4	130 ± 8.5	127 ± 6.0	0.5302
Total EAA	311 ± 23.9	305 ± 17.4	317 ± 39.5	301 ± 12.3	289 ± 10.7	0.6521
Total amino acids	565 ± 45.7	555 ± 30.0	575 ± 65.2	556 ± 14.4	529 ± 28.8	0.7145

Abbreviations: BCAA, branched-chain amino acid; EAA, essential amino acid; P3, mixture of 3 microbial protease preparations.

1 Data are mean ± SD (*n* = 3 per group).

2 Significant differences between groups are denoted by unshared lower-case letters (a, b, c).

### Clinical trial

#### Appetite VAS and GI symptoms

**FIGURE 2:**
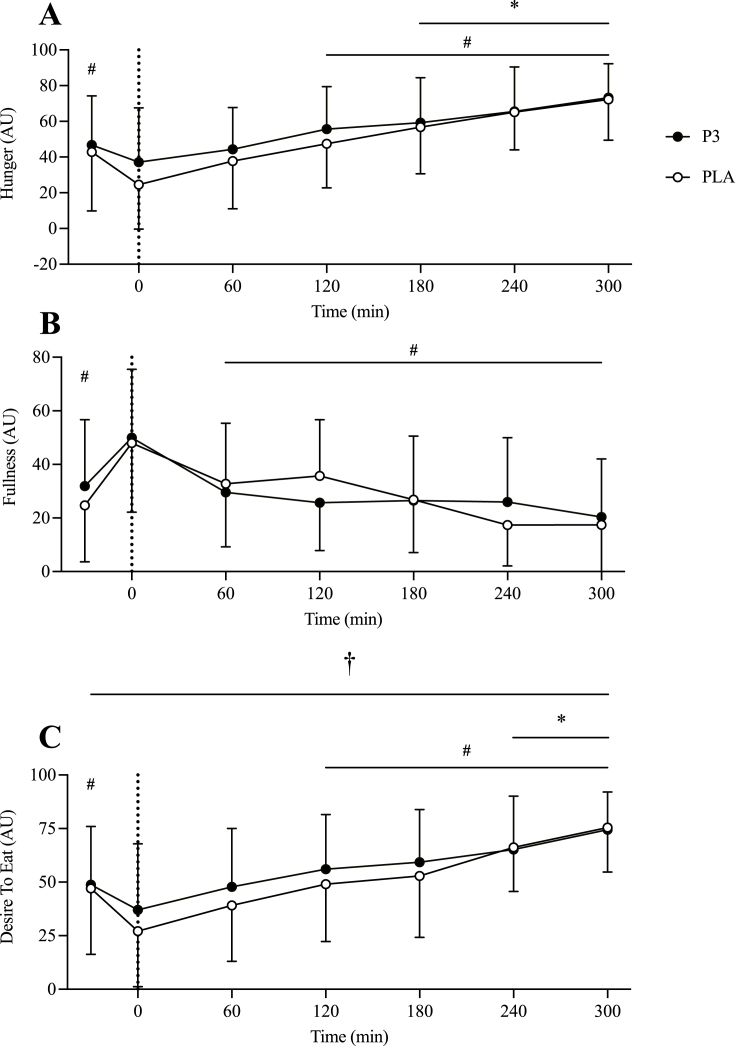
Self-reported feelings of hunger (A), fullness (B), and desire to eat (C) during the postabsorptive and postprandial period. Ingestion of the mixture of 3 microbial protease preparations (P3; *n* = 24) or placebo (PLA; *n* = 24) is denoted by the vertical dotted line. Values are means ± SD. ∗Denotes significant difference from baseline (*t* = –5); #Denotes significant difference from immediately post-ingestion of P3 or PLA (*t* = 0). †Denotes significant main effect of condition (*P* < 0.05).

Participants’ baseline characteristics and dietary intake are shown in Table 3. Self-reported feelings of hunger decreased after protein ingestion and increased over time (*P* < 0.001) with a trend for greater hunger for P3 than for PLA [3.219 (–0.10, 6.54) AU, *P* = 0.058; Figure 2A]. Self-reported feelings of fullness increased after protein ingestion and decreased over time (*P* < 0.001) with no difference between conditions [1.37 (–2.60, 5.35) AU, *P* = 0.497; Figure 2B]. Self-reported desire to eat decreased after protein ingestion and increased over time (*P* < 0.001) with a higher desire to eat for P3 than for PLA [3.94 (0.38, 7.50) AU, *P* = 0.030; Figure 2C]. There was no significant difference in self-reported GI symptoms between conditions (*P ≥* 0.480, data not shown). The only symptoms that were reported were nausea (*n* = 3), urge to vomit (*n* = 1), bloating (*n* = 3), and flatulence (*n* = 4). Collectively these symptoms ranged in severity from 1 to 5 on a 10-point scale, where 10 is most severe (average 2.8 ± 1.5).

**TABLE 3:** Baseline characteristics of trial participants

Variable	Male (*n* = 12)	Female (*n* = 12)	Total (*n* = 24)
Age (y)	27 ± 5	26 ± 3	27 ± 4
Body mass (kg)	83.0 ± 8.1	66.3 ± 6.7	74.6 ± 11.2
BMI (kg/m)	25.4 ± 2.0	24.2 ± 1.7	24.8 ± 1.9
Systolic blood pressure (mmHg)	116 ± 13	121 ± 11	119 ± 12
Diastolic blood pressure (mmHg)	67 ± 9	67 ± 6	67 ± 8
Body fat (%)	21.6 ± 5.8	36.8 ± 5.3	29.2 ± 9.4
Lean body mass (kg)	61.9 ± 8.6	38.9 ± 4.5	50.4 ± 13.5
Fasting glucose (mmol⋅L^-1^)	4.96 ± 0.52	4.84 ± 0.51	4.90 ± 0.52
Energy intake (kcal·d^-1^)	2,369 ± 562	2,009 ± 585	2,189 ± 595
Relative protein intake (g·kg^-1^·d^-1^)	1.5 ± 0.4	1.4 ± 0.7	1.5 ± 0.6
Carbohydrate Intake (g·d^-1^)	252 ± 64	221 ± 73	236 ± 69
Fat intake (g·d^-1^)	102 ± 27	84 ± 24	93 ± 27

Data are mean ± SD.

#### Plasma glucose and insulin analyses

**FIGURE 3:**
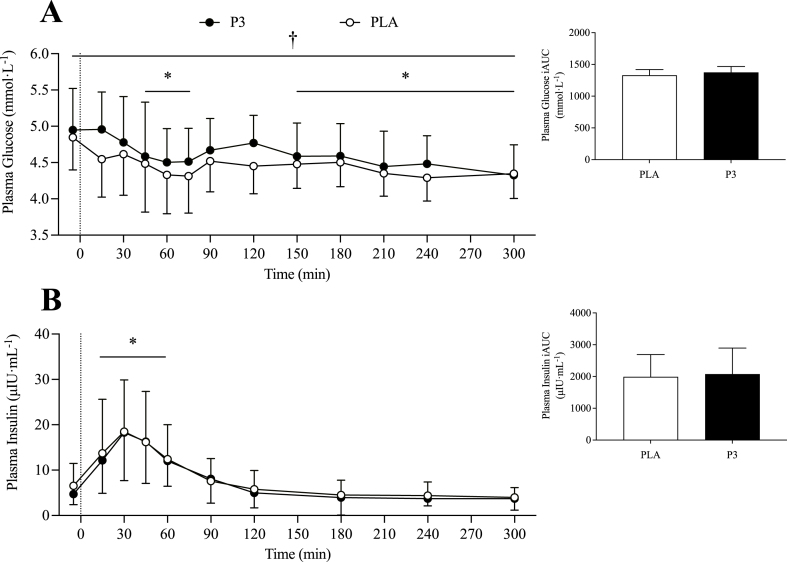
Plasma glucose (A) and insulin (B) concentrations during the postabsorptive and postprandial period. Insets show the incremental area under the curve (iAUC) over the entire (0‒5 h) postprandial period. Ingestion of the mixture of 3 microbial protease preparations (P3; *n* = 24) or placebo (PLA; *n* = 24) is denoted by the vertical dotted line. Values are means ± SD. ∗Denotes significant difference from baseline *(t* = –5; *P* < 0.05). †Denotes significant main effect of condition (*P* < 0.05).

Plasma glucose concentrations decreased after protein ingestion (*P* < 0.001) with higher glucose concentrations for P3 than for PLA [2.94 (1.82, 4.05) mmol·L^-1^, *P* < 0.001; Figure 3A]. In accordance, plasma glucose iAUC was 4.4% higher for P3 during the early (0‒2 h) postprandial period compared with PLA [24.7 (0.7, 48.7) mmol·L^-1^, *P* = 0.045], with no significant difference between conditions during the total postprandial period [50.1 (–17.3, 117.4) mmol·L^-1^, *P* = 0.136].

Plasma insulin concentrations increased after protein ingestion (*P* < 0.001) with no difference between conditions [–0.55 (–1.66, 0.56) μIU·mL^-1^; *P* = 0.331; Figure 3B]. Plasma insulin concentrations iAUC did not differ between conditions during both the early [13.73 (–244.0, 271.4) μIU·mL^-1^; *P* = 0.907] and total [143.29 (–382.4, 669.0) μIU·mL^-1^; *P* = 0.547] postprandial periods.

#### Plasma amino acid analyses

**FIGURE 4:**
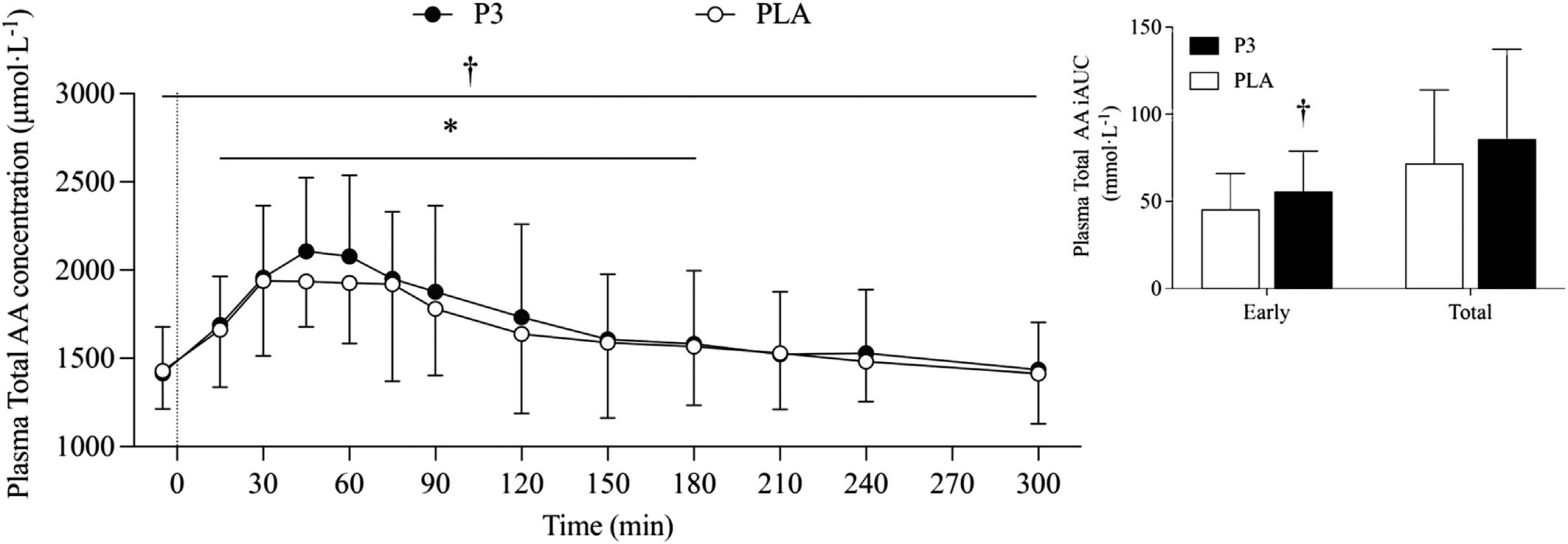
Plasma total amino acid concentrations during the postabsorptive and postprandial period. Insets show the incremental area under the curve (iAUC) over the early (0‒2 h) and total (0‒5 h) postprandial periods. Ingestion of the mixture of 3 microbial protease preparations (P3; *n* = 24) or placebo (PLA; *n* = 24) is denoted by a vertical dotted line. Values are means ± SD. ∗Denotes significant difference from baseline (*t* = –5; *P* < 0.05); †Denotes significant difference between conditions (*P* < 0.05).

There were no differences in peak plasma amino acid concentration (*C*_max_) or time to peak plasma amino acid concentration (*T*_max_) for leucine, BCAA, EAA, or TAA (see Supplemental Table 1). Plasma TAA concentration increased after protein ingestion (*P* < 0.001) and returned to baseline values at *t* = 180 min, with higher TAA concentrations for P3 when compared with PLA [52.1 (12.8, 91.4) μmol·L^-1^; *P* = 0.010; Figure 4]. Plasma TAA iAUC was 22.3% greater for P3 compared with PLA during the early postprandial period [11.08 (2.88, 19.29) mmol·L^-1^, *P* = 0.010], with no significant difference during the total postprandial period [18.38 (–8.04, 44.79) mmol·L^-1^; *P* = 0.164].

**FIGURE 5:**
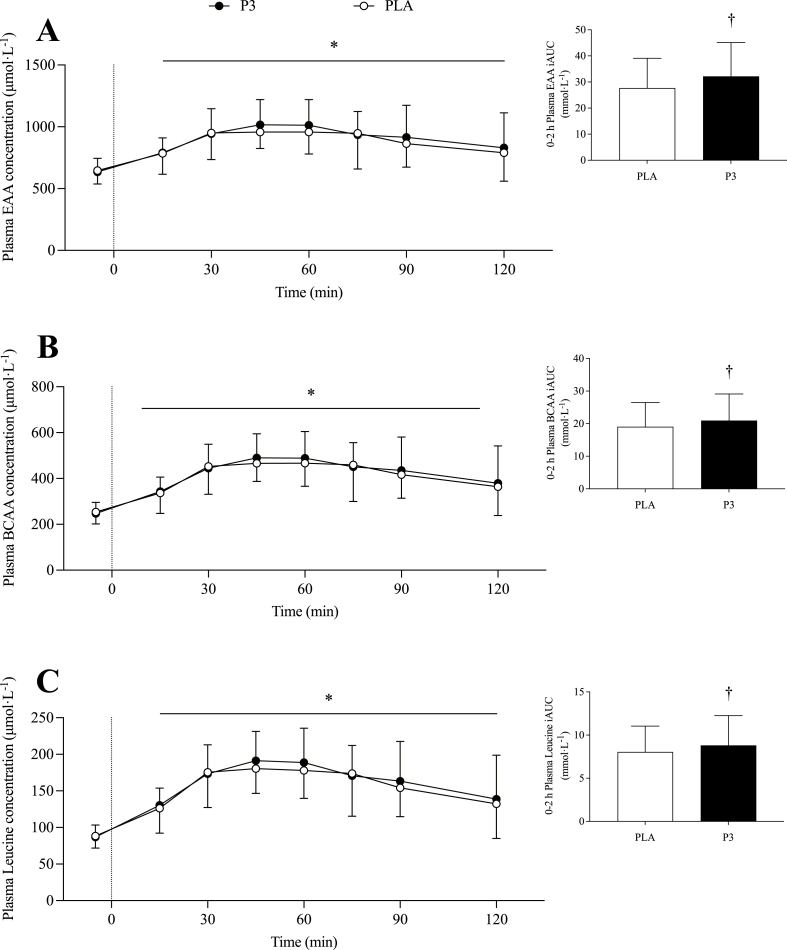
Plasma essential amino acid (A), BCAA (B), and leucine (C) concentrations during the postabsorptive and postprandial periods. Insets show the incremental area under the curve (iAUC) over the early (0‒2 h) postprandial period. Ingestion of the mixture of 3 microbial protease preparations (P3; *n* = 24) or placebo (PLA; *n* = 24) is denoted by the vertical dotted line. Values are means ± SD. ∗Denotes significant difference from baseline (*t* = –5; *P* < 0.05); †Denotes significant difference between conditions (*P* < 0.05).

Plasma EAA concentration increased after P3 and PLA ingestion (*P* < 0.001) and returned to baseline at *t* = 240 min, with a marginally significant difference between conditions [16.6 (–3.1, 36.3) μmol·L^-1^; *P* = 0.099; data not shown]. Plasma EAA iAUC was 16.2% greater for P3 compared with PLA during the early postprandial period [4.70 (0.90, 8.51) mmol·L^-1^, *P* = 0.017; Figure 5A], with no difference between conditions during the total postprandial period [8.77 (–3.60, 21.13) mmol·L^-1^, *P* = 0.156; data not shown].

Plasma BCAA concentration increased after protein ingestion (*P* < 0.001) and returned to baseline at *t* = 300 min, with no difference between conditions [4.6 (–6.4, 15.7) μmol·L^-1^; *P* = 0.410; data not shown]. Plasma BCAA iAUC was 9.9% greater for P3 compared with PLA during the early postprandial period [1.98 (0.14, 3.81) mmol·L^-1^, *P* = 0.036; Figure 5B], with no difference across the total postprandial period [3.35 (–2.03, 8.72) mmol·L^-1^, *P* = 0.210; data not shown].

Plasma leucine concentration increased after protein ingestion (*P* < 0.001) and remained elevated above baseline until *t* = 300 min, with no difference between conditions [2.4 (–2.0, 6.9) μmol·L^-1^, *P* = 0.282; data not shown]. Early plasma leucine iAUC was 9.4% higher after P3 ingestion compared with PLA [0.80 (0.13, 1.46) mmol·L^-1^; *P* = 0.021; Figure 5C], with no difference between conditions for the total postprandial period[1.03 (–0.89, 2.95) mmol·L^-1^, *P* = 0.277; data not shown].

## Discussion

Proteolytic enzyme supplementation is emerging as a potential strategy for augmenting amino acid bioavailability after dietary protein consumption [13,14]. We investigated this hypothesis in a crossover trial by examining the postprandial effects of consuming pea protein with OPTIZIOME® P³ HYDROLYZER® a mixture of 3 microbial protease preparations. We demonstrated that P3 co-ingestion with pea protein resulted in significantly higher TAA concentrations over time, as well as significantly greater net plasma exposure (iAUC) to leucine, BCAAs, EAAs, and TAAs within the early (0‒2 h) postprandial period compared with pea protein with placebo.

There is an evolving emphasis on consuming plant-based foods within the context of a healthy dietary pattern [31]. Beyond manufacturing optimization, various dietary strategies have been used to improve the bioavailability of plant-based proteins, such as pairing complementary proteins together or increasing total protein intake [9,32]. As an alternative strategy, we tested whether microbial protease co-ingestion with pea protein could improve protein bioavailability in young, healthy adults. Theoretically, supplemental microbial proteases work alongside endogenous proteases (e.g., pepsin, trypsin, chymotrypsin) to improve peptide and amino acid release from dietary protein, a notion supported by in vitro digestion simulation results [16] (Table 2) and a limited number of published clinical trials [13,33]. In our clinical trial, we demonstrated a condition effect for total plasma amino acid concentrations with higher concentrations observed for P3 supplementation compared with PLA during the 0‒5 h postprandial period. Furthermore, although the 0‒5 h postprandial concentrations for plasma leucine, BCAA, and EAA did not differ significantly between P3 and PLA, the early net exposure (0‒2 h) to leucine, BCAA, EAA, and TAA were all potentiated with P3 compared with PLA. This early amino acid release is practically relevant considering how performance nutrition strategies often pursue such rapid aminoacidemia to support the immediate postexercise whole body and muscle adaptive responses [6,29,34,35]. Moreover, it has been shown that enhancing plasma amino acid availability by food fortification techniques is relevant within clinical feeding formulas to augment the anabolic response [36]. Beyond amino acids, it is worth noting that we also demonstrated higher postprandial plasma glucose concentrations in the P3 compared with the PLA trial. This effect is not surprising because P3 is a mixture of wild-type enzyme preparations with “side activities” that may include amylase and glucoamylase activities capable of digesting the modest carbohydrate fraction of pea protein isolate. Overall, and considering the previously discussed interest in plant-based proteins, improving the digestibility of less traditional proteins such as the pea protein used in this trial is a notable opportunity for enhancing both performance and clinical nutrition outcomes.

Previous attempts to determine the effects of protease co-supplementation with dietary protein in humans have yielded mixed results [13,14]. For example, Townsend et al. [14] demonstrated no statistically significant effect of co-ingesting a commercial mixture of a bacterial protease and bromelain with 26 g whey protein on the postprandial rise in plasma amino acids during recovery from resistance exercise in young adult men. Comparatively, P3’s efficacy in this trial could be attributed to higher overall proteolytic activity per dose and robust activity at usual gastric pH as informed by the in vitro oro-gastric digestion simulation. Alternatively, the discrepancy with the current results may relate to the protein source tested. For example, although pea protein isolate has demonstrated high amino acid ileal digestibility, and when combined with the amino acid composition, resulted in a DIAAS of 1.00) [8], whey protein has DIAAS > 1.00 and more rapid *T*_max_ in aminoacidemia trials [[37], [38], [39]]. Such differences in ileal digestibility/amino acid composition and an already rapid release of postprandial amino acids into circulation may have limited the ability of a protease supplement to further improve the digestion of whey protein and thus contribute to the lack of effect observed in that comparative study. Moreover, the inclusion of exercise may have also played a role in the lack of effect considering exercise is known to modulate gut permeability [35,40]. Nonetheless, a second human trial by Oben et al. [13] did show higher amino acid concentrations following co-ingestion of a 2.5 g mixture of fungal proteases with 50 g whey protein concentrate in young healthy male participants. However, the continuous postprandial aminoacidemia observed over 4 h was inconsistent with typical protein absorption kinetics [13]. The lack of clear congruence between our findings demonstrating the clinical postprandial aminoacidemia efficacy of P3 and these 2 related studies utilizing co-ingestion with whey protein underscores the need for additional research in the area, particularly that which is focused on dietary proteins of lower digestibility.

Secondarily, we also examined subjective appetite VAS and GI symptom responses. For the appetite measurements (i.e., hunger, fullness, and desire to eat), only participants’ self-reported desire to eat was significantly different between conditions. Participants consuming pea protein with P3 showed a higher postprandial desire to eat compared with pea protein with PLA. This difference may reflect the greater speed and efficiency of protein digestion with protease supplementation, thereby inducing a greater desire for further caloric intake, perhaps through the sensing of short-chain peptides and free amino acids. Regarding GI tolerance, self-reported GI symptoms were few and no different between P3 and PLA groups, in agreement with a prior study showing that pea protein is well tolerated [41]. These exploratory appetite and GI outcomes serve as a useful foundation for future studies.

Despite showing a significant acute increase in postprandial plasma amino acid concentrations with a plant-based protein source with high amino acid ileal digestibility, this clinical study comes with several limitations, including limited generalizability to other protein sources with differential ileal digestibility and/or amino acid compositions, and lack of whole-body protein kinetic measurements to define the metabolic fate of the elevated circulating amino acids. It is also important to acknowledge that our findings are acute metabolic findings and thus future work is required to define the longer-term clinical significance such as improved quantity/quality of muscle protein and physical performance outcomes with more prolonged supplementation. Concerning the in vitro research, measurement of free amino acid release in the static oro-gastric digestion simulation proved useful for optimizing the P3 protease mixture and delivering an effective formulation for the clinical trial. However, the in vitro static oro-gastrointestinal digestion simulation showed no differences between P3 treatment and pancreatin control, suggesting that a semidynamic protocol to better model pancreatin output and transit of chyme is warranted.

In conclusion, we provide important insights into the acute effects of microbial protease supplementation on modulating postprandial plasma amino acid concentrations after consumption of plant protein. We demonstrated that consuming a mixture of 3 microbial proteases alongside 25 g of pea protein isolate resulted in significantly higher TAA concentrations over the total 5-h postprandial testing window, as well as significantly greater net plasma exposure (iAUC) to leucine, BCAAs, EAAs, and TAAs within the early (0‒2 h) postprandial period compared with consuming pea protein with placebo in healthy adults. Future studies, however, are warranted to define the metabolic fate (i.e., synthesis/oxidation) of changes in plasma amino acid concentrations after microbial protease supplementation and the long-term clinical relevance with more chronic supplementation.

## Author contributions

The authors’ responsibilities were as follows – NAB, KJMP, SMG: contributed to the conception and design of the experiments; MTD, KJMP, SMG, ATA, NAB: contributed to drafting or revising the intellectual content of the manuscript and had primary responsibility for the final content; KJMP, ATA, RMK, MTD, SAP: contributed to the collection of data. KJMP, ATA, MTD, NAB, JLG, CFM, RMK, TMB, AVU, LLB, RND, SMG, KMT: contributed to the analysis and interpretation of data; and all authors: read, edited, and approved the final version of the manuscript.

### Conflict of interest

This project was funded by BIO-CAT, Inc. SMG, JLG, and KMT are employees of BIO-CAT, Inc., which provided the investigational products and distributes OPTIZIOME® P3 HYDROLYZER®. BIO-CAT, Inc. filed a patent application related to the enclosed findings (International Patent Application No. PCT/US2022/022053). The funders were involved in the design of the study, in the collection of in vitro data only, in the interpretation of data, in the writing of the manuscript, and in the decision to publish the results. The authors declare no other conflicts of interest.

### Funding

This study was funded by BIO-CAT, Inc.

### Data availability

Data described in the manuscript, code book, and analytic code will be made available upon request pending approval from the principal investigator.
