# Supplementary material for: Acute Microbial Protease Supplementation Increases Net Postprandial Plasma Amino Acid Concentrations After Pea Protein Ingestion in Healthy Adults: A Randomized, Double-Blind, Placebo-Controlled Trial
Source: J Nutr. 2024 Mar 11;154(5):1549–60. doi: 10.1016/j.tjnut.2024.03.009 (PMC11130700; doi:10.1016/j.tjnut.2024.03.009)

| **Supplemental Table 1.** Peak plasma amino acid concentration (C_max_) and time-to-peak plasma amino acid concentration (T_max_) following co-ingestion of 20 g protein and P3 or PLA. | | | | | | | | |
| --- | --- | --- | --- | --- | --- | --- | --- | --- |
|  |  | C_max_ (mmol·L^-1^) | |  |  | T_max_ (min) | |  |
|  |  | P3 | PLA | *P*-value |  | P3 | PLA | *P*-value |
| Leucine |  | 212.7 ± 52.5 | 208.0 ± 59.4 | 0.607 |  | 54.4 ± 17.0 | 53.1 ± 18.8 | 0.811 |
| BCAA |  | 547.6 ± 144.5 | 540.0 ± 158.2 | 0.746 |  | 60.0 ± 20.7 | 53.1 ± 18.8 | 0.241 |
| EAA |  | 1119.7 ± 256.3 | 1094.6 ± 281.8 | 0.537 |  | 61.9 ± 27.4 | 58.8 ± 34.2 | 0.726 |
| TAA |  | 2295.5 ± 489.8 | 2232.1 ± 541.6 | 0.394 |  | 61.9 ± 27.4 | 67.5 ± 49.8 | 0.608 |
| BCAA, branched-chain amino acids; EAA, essential amino acids; TAA, total amino acids | | | | | | | | |

**Supplemental Methods**

**Incremental Area Under the Curve (iAUC) Calculations**

Postprandial iAUC was calculated geometrically using a trapezoidal method that ignored area under the baseline using the equation below.


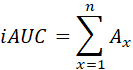


For the first time interval (i.e., $x=1$):

if $G_{1}>G_{0}$, A_1_ = $\left( G_{1}-G_{0} \right)\times(t_{1}-t_{0})/2$;

otherwise, $A_{1}=0$.

For other time intervals (i.e., $x>1$):

if $G_{x}\geq G_{0}$and $G_{x-1}\geq G_{0}$, $A_{x}={(((G}_{x}- G_{0})/2)+ {(G}_{x-1}- G_{0})/2)\times{(t}_{x}-t_{x-1})$;

if $G_{x}\geq G_{0}$and $G_{x-1}< G_{0}$, $A_{x}={((G}_{x}- G_{0})^{2}/{(G}_{x}- G_{x-1}))\times{(t}_{x}-t_{x-1})/2)$;

if $G_{x}<G_{0}$and $G_{x-1}\geq G_{0}$, $A_{x}={((G}_{x-1}-G_{0})^{2}/{(G}_{x-1}- G_{x}))\times{(t}_{x}-t_{x-1})/2)$;

if $G_{x}<G_{0}$and $G_{x-1}< G_{0}$, $A_{x}=0$.

where:

t: time (min)

G_x_: blood metabolite concentration

A_x_: the AUC for the x^th^ time interval between t_x-1_ and t_x_.

For each participant, iAUC was calculated for t = 120 and 300 min.

**Supplemental Figure 1.** CONSORT flow diagram.


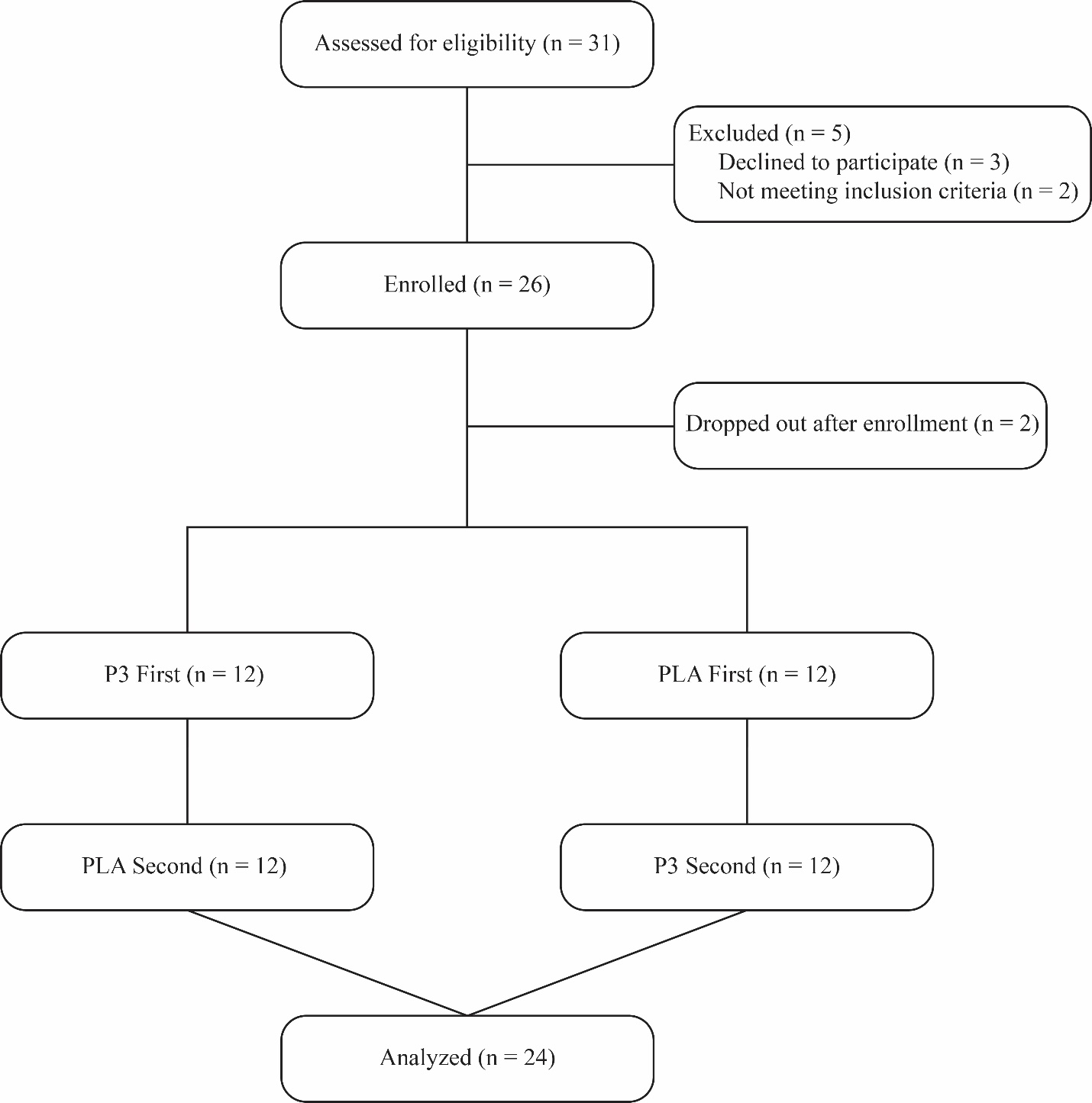

Supplement: Multimedia component 1 [file mmc1.docx]
